# Distance to care, enrollment and loss to follow-up of HIV patients during decentralization of antiretroviral therapy in Neno District, Malawi: A retrospective cohort study

**DOI:** 10.1371/journal.pone.0185699

**Published:** 2017-10-03

**Authors:** Alyssa Bilinski, Ermyas Birru, Matthew Peckarsky, Michael Herce, Noel Kalanga, Christian Neumann, Gay Bronson, Stephen Po-Chedley, Chembe Kachimanga, Ryan McBain, James Keck

**Affiliations:** 1 Abwenzi Pa Za Umoyo, Neno, Malawi and Partners In Health, Boston, Massachusetts, United States of America; 2 Interfaculty Initiative in Health Policy, Harvard Graduate School of Arts and Sciences, Cambridge, Massachusetts, United States of America; 3 Division of Infectious Diseases, Department of Medicine, University of North Carolina School of Medicine, Chapel Hill, North Carolina, United States of America; Tulane University School of Public Health and Tropical Medicine, UNITED STATES

## Abstract

HIV/AIDS remains the second most common cause of death in low and middle-income countries (LMICs), and only 34% of eligible patients in Africa received antiretroviral therapy (ART) in 2013. This study investigated the impact of ART decentralization on patient enrollment and retention in rural Malawi. We reviewed electronic medical records of patients registered in the Neno District ART program from August 1, 2006, when ART first became available, through December 31, 2013. We used GPS data to calculate patient-level distance to care, and examined number of annual ART visits and one-year lost to follow-up (LTFU) in HIV care. The number of ART patients in Neno increased from 48 to 3,949 over the decentralization period. Mean travel distance decreased from 7.3 km when ART was only available at the district hospital to 4.7 km when ART was decentralized to 12 primary health facilities. For patients who transferred from centralized care to nearer health facilities, mean travel distance decreased from 9.5 km to 4.7 km. Following a transfer, the proportion of patients achieving the clinic’s recommended ≥4 annual visits increased from 89% to 99%. In Cox proportional hazards regression, patients living ≥8 km from a health facility had a greater hazard of being LTFU compared to patients <8 km from a facility (adjusted HR: 1.7; 95% CI: 1.5–1.9). ART decentralization in Neno District was associated with increased ART enrollment, decreased travel distance, and increased retention in care. Increasing access to ART by reducing travel distance is one strategy to achieve the ART coverage and viral suppression objectives of the 90-90-90 UNAIDS targets in rural impoverished areas.

## Introduction

HIV/AIDS remains a leading cause of death and disability, with over 35 million people infected worldwide, resulting in 1.5 million deaths annually [1]. Although the Millennium Development Goals aimed for universal access to antiretroviral therapy (ART) by 2010 [2], in 2013, only a third of those eligible for ART living in low- and middle-income countries (LMICs) received it [3,4]. In Africa, an estimated 66% of eligible people living with HIV were not receiving ART in 2013 [5].

In order to increase access to HIV treatment, many countries in Sub-Saharan African (SSA) have decentralized HIV care from hospitals to health centers and other primary health facilities closer to the community [6]. Decentralization seeks to reduce distance traveled by patients, task shift ART initiation and HIV management from physicians to lower-cadre health workers, and integrate delivery of ART within existing primary health care systems.

Malawi, a country of over 16 million people with an adult HIV prevalence of 10% and 84% of its population living in rural areas, has prioritized ART decentralization, achieving ART coverage levels of 80% in 2013 [7–9]. Neno District is one of the most impoverished and geographically isolated regions of Malawi. In Neno District, universal HIV testing became available in 2007, and ART care was decentralized from one district hospital to 12 health facilities between 2006 and 2012, with nurses and non-physician clinicians assuming primary responsibility for ART service delivery.

In Malawi and elsewhere in SSA, ART decentralization has generally improved outcomes [6,10]. Implementation studies from Malawi, South Africa, and Ethiopia have demonstrated that decentralization leads to improved patient retention and enrollment [10], [11], particularly for stable adult patients on ART [12]. Decentralization has also been shown to reduce costs by shifting care to lower-level professionals [13], and to improve access to care for populations from lower socioeconomic strata [14].

Despite the available evidence, gaps remain in our understanding of the effects of initiating ART at decentralized facilities [6,10,15] and the associations between this kind of full decentralization and longitudinal changes in patient behavior and health outcomes [10]. To address these evidence gaps, we used a geographic information system (GIS) and an electronic medical record (EMR) to track changes in patients’ travel distance, treatment-seeking behavior, and care retention from 2006 to 2013 during ART decentralization in rural Neno District, Malawi.

## Materials and methods

### ART decentralization and service delivery in Neno

**Fig 1:**
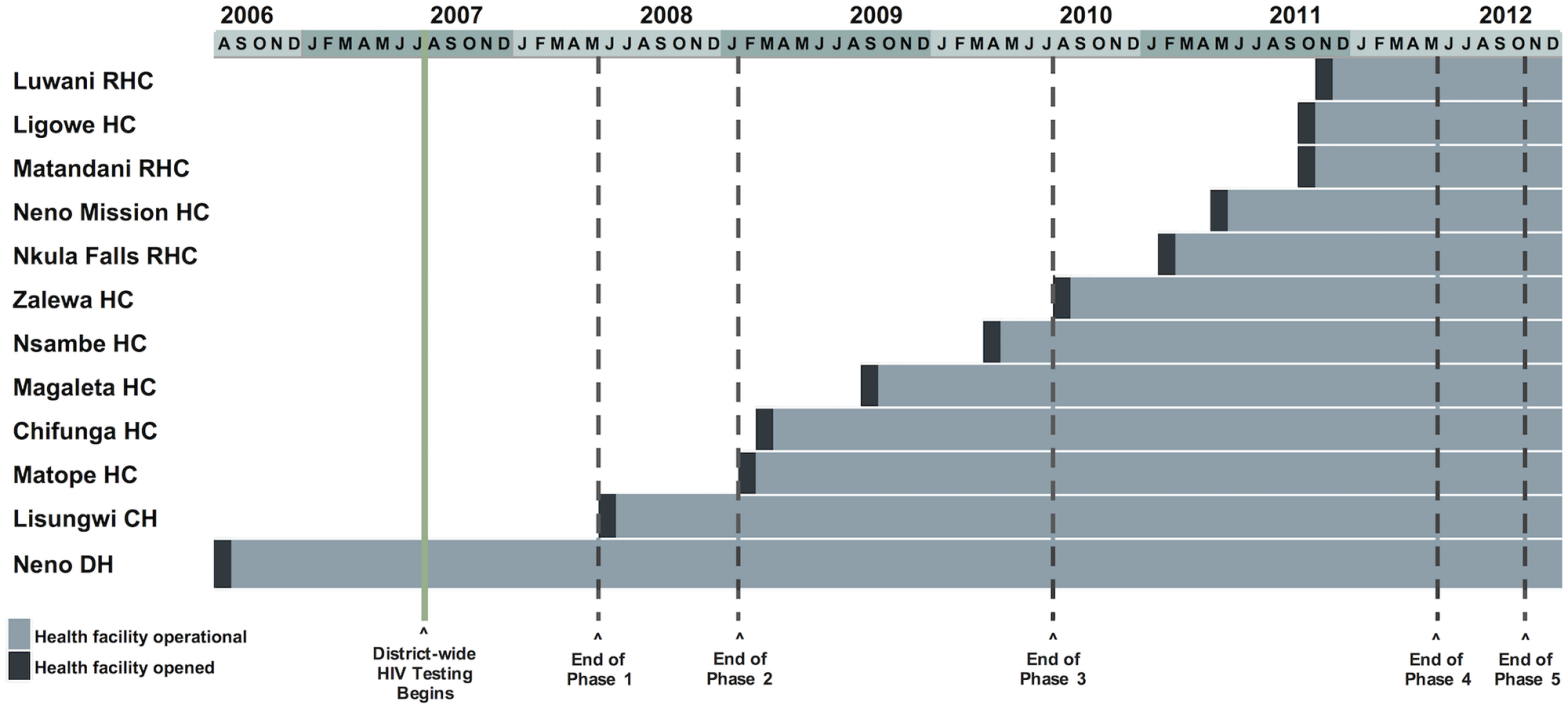
ART decentralization timeline. The timeline displays HIV care in Neno District from August 1, 2006, when public care first became available, through December 31, 2012. (DH = district hospital, CH = community hospital, HC = health center, RHC = rural health center).

We conducted our study in Neno District, Malawi, a mountainous rural region of 1469 km^2^ in the south of the country with a 2013 population of approximately 137,000 people [16]. In 2007, Partners In Health/Abwenzi Pa Za Umoyo (PIH/APZU) began a partnership with the Ministry of Health (MOH) to provide comprehensive, community-based health care in Neno District. PIH/APZU utilizes an accompaniment model to pair community health workers with persons living with HIV (PLWH), which has successfully promoted patient retention and favorable clinical outcomes in several resource-constrained settings, including Neno [17,18]. Between 2006 and the end of 2012, the MOH opened 10 static ART clinics supported by PIH/APZU at primary health facilities throughout the district to decentralize front-line HIV care (Fig 1). When new ART clinics opened, all patients were invited to transfer to an ART clinic closer to their home.

HIV treatment at Neno District facilities followed national guidelines [19,20]. From 2006 to 2010, HIV-infected patients were ART eligible if they met one of the following criteria: CD4 count <250 cells/mm^3^, WHO Stage 3 or 4 disease, or Stage 2 disease with a total lymphocyte count <1,200 cells/ mm^3^. In 2011, Malawi introduced the following new guidelines: an increased CD4 count threshold for ART initiation (350 cells/mm^3^); initiation of universal lifelong ART for all HIV-infected children under 2 years; and Option B+, which included lifelong ART for all HIV-infected pregnant and breastfeeding women. Patients who initiated ART visited a health facility every 1–3 months for medical review and to refill their supply of cotrimoxazole preventative therapy and ART.

### Study design and population

We performed a retrospective cohort study of all adult and pediatric patients living in Neno District who initiated ART between August 1, 2006 and December 31, 2013 at any clinic in the district and had at least two ART visits in this time period.

### Data source

Malawi’s national HIV program uses a paper-based reporting system to collect data on patient demographics and service provision. In Neno, PIH/APZU augments the MOH system by transcribing the paper patient data into equivalent electronic forms in an electronic medical record (EMR) using the OpenMRS platform [21]. To monitor and ensure EMR data fidelity the PIH/APZU informatics team operated a data quality assessment system. It included logic checks built into EMR, automated data quality reports, and routine cross-referencing of aggregate EMR data. Key indicators were reviewed on weekly, monthly and quarterly bases, including the quarterly Malawi Ministry of Health HIV program data audit reports. In addition, a closed feedback loop between the clinics and the EMR was enforced to ensure that findings were shared in a timely manner in both directions. We exported individual-level data, including health facility visit records, and ascertained patient transfers between health facilities using a unique EMR identifier.

### Geospatial data collection and analysis

We collected GPS coordinates with a GPSMAP 60CSx device (Garmin, Olathe, Kansas, USA) for each Neno health facility and each village recognized by the government of Malawi. To ensure patient privacy and simplify the data collection process, we collected coordinates at the commercial center of each village, or if no commercial center existed, at the home of the village chief. GPS coordinates were converted to a shapefile using DNR Garmin version 6. For each patient, we linked their home village reported at ART registration to corresponding GPS coordinates. In our analyses, we excluded patients with missing village information.

For travel distance analyses, we used both Euclidean distance and cost distance. Euclidean measurements calculated distance “as the crow flies”, without regard to topography. Cost distance considered topographical features when calculating distance between two points. To calculate cost distance, we weighted travel distance by topographical slope. The cost distance between two points was minimum weighted distance between two points. Distance analyses were performed in ArcMap 10.2 and Python 2.7.

### Statistical analysis

To analyze the impact of full ART decentralization, we divided Neno district’s decentralization process into five phases based on the following rationale: during phase 1 ART was available only at the Neno District Hospital; in phase 2 the Lisungwi Community Hospital began to provide ART. During phases 3 and 4, ART became available at primary health facilities, with 4 facilities providing ART during phase 3 and an additional 6 during phase 4. Phase 5 captured the six-month period immediately following full ART decentralization (Fig 1). The ends of the first three phases (31 May 2008, 31 January 2009, and 31 July 2010) correspond to the day prior to the opening of a hospital or clinic in the next wave of service decentralization. The fourth phase ended on 30 April 2012, six months after the final health facility began providing HIV treatment, and the final phase ended on 31 October 2012, 6 months thereafter. The fourth phase also captured the 2011 introduction of Option B+ and guideline shift.

Our primary outcome measures were appointment adherence and loss to follow up. Appointment adherence is thought to provide a measure of patient engagement in HIV care, and may predict viral suppression [22,23]. Because the EMR lacked consistent information on the quantity of ARVs dispensed at each visit (which varied from a one- to three-month supply), we were unable to calculate when the patient’s next visit should occur. Instead we estimated appointment adherence by applying the MOH guideline that all patients have at least 4 visits per year, suggesting that those with fewer visits would have experienced ART interruption. Therefore, we conservatively calculated the median number of visits per year for each patient, and defined patients with fewer than 4 visits per year to be “non-adherent” to appointments.

We defined a patient as “lost to follow up (LTFU)” if his or her EMR status was not “alive and on ART.” This included patients whose vital status was recorded as dead, defaulted, stopped or transferred out (without a corresponding “transfer in” to a different facility) or if the patient did not have ≥1 recorded ART visit with 180 days of the relevant time point, a measure of LTFU previously validated in SSA [24]. We included death and default within our definition of LFTU to account for previously described high mortality among patients who leave care in SSA [25]. However, due to our inability to track people who transferred outside of the district who may have received care, this approach likely resulted in a conservative estimate of patient retention. Non-LTFU patients were considered “active.”

We report descriptive statistics for patients organized by decentralization phase, including the number of patients enrolled in care at any point during each phase, as well as the number of patients newly enrolled during each phase. We describe the sub-cohorts of patients that newly initiated ART for each phase and present their demographic, clinical, and distance information. We defined a patient as “in care” at the end of a phase if his/her most recent documented status prior to the end of the phase was “alive and on ART” and if he/she had ≥1 ART visit within 180 days of the phase date [24].

We examined changes in appointment adherence and LTFU one year after enrollment before and after transfer in the subgroup of patients who transferred at least once during the study period, matched by patient (before/after transfer), with repeated measures analysis of variance (ANOVA). We also used t-tests to compare demographic characteristics, annual visits and LTFU between patients who transferred and those who never transferred.

Finally, to assess the association between travel distance and LTFU, we used Cox proportional hazards regression to estimate the hazard ratio associated with being LTFU as a function of travel distance. We dichotomized travel distance to care at 8km based on Malawi’s definition of access to care [26] and treated it as a time-varying covariate to control for length-biased sampling, i.e. that patients closer to care were often enrolled for longer periods of time [27,28]. We estimated unadjusted odds ratios and odds ratios adjusted for potential confounders, all of which remained constant over time, except for whether a patient had transferred in a particular enrollment period. Statistical analyses were performed in R 3.1.0.

### Ethics statement

The Partners Healthcare Human Research Committee, USA (Protocol #: 2014P001460) and the National Health Sciences Research Committee of Malawi (Protocol #: 1216) granted ethical approval for this study.

## Results

Overall, 5,969 unique patients enrolled into ART care at a Neno District health facility between August 1, 2006 and November 1, 2012, and attended at least 2 visits. Of these patients, 753 (13%) reported home villages outside of Neno District, 301 (5%) were missing valid village information, and 50 (<1%) lived in Neno District villages for which we lacked GPS data. Therefore, 4,865 unique patients (82%) ever enrolled in the ART program on or before November 1, 2012 with at least 2 visits, 1,074 (22%) of whom transferred from one health facility to another at least once during the study period.

### Phase analysis

**Fig 2:**
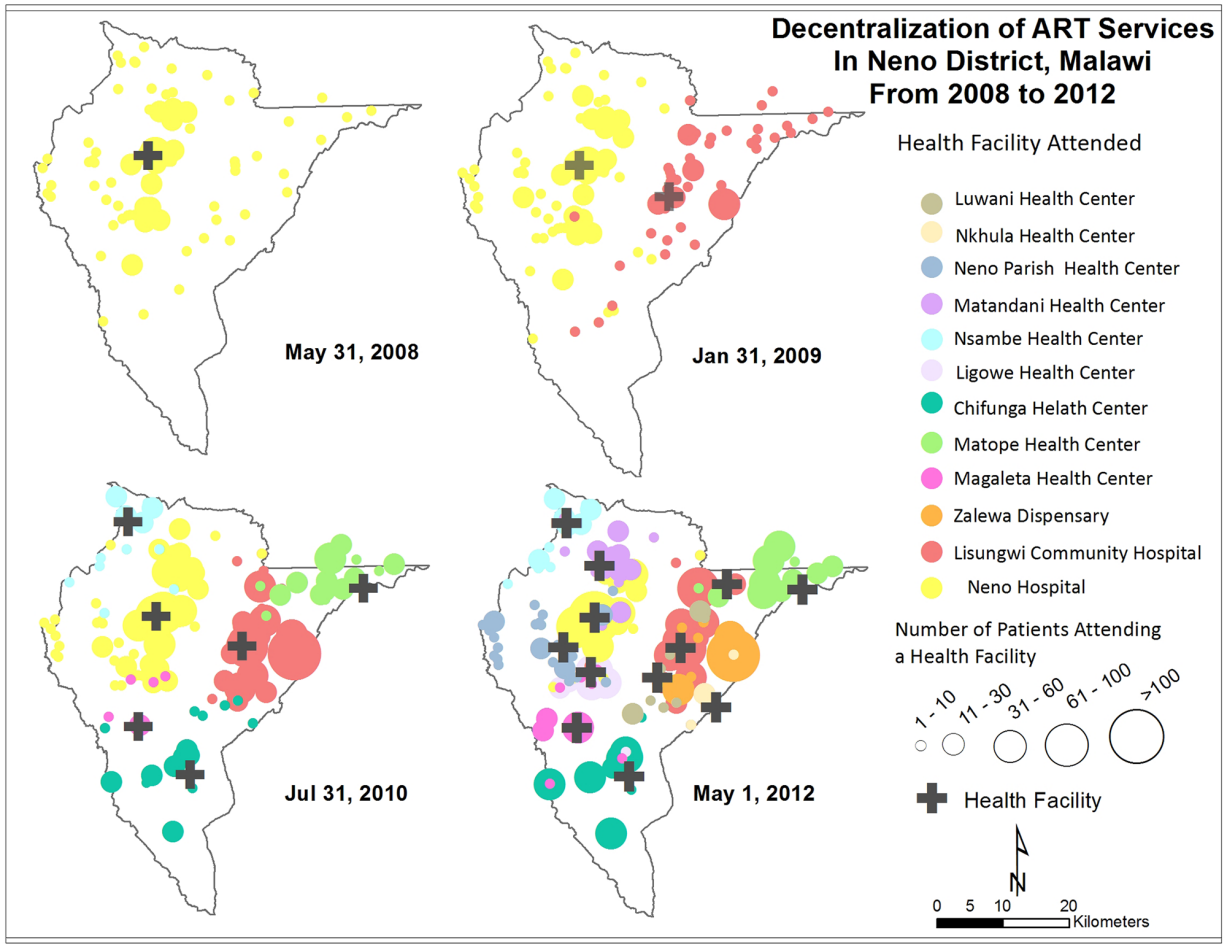
Map of decentralization of ART services from 2008 to 2012. Crosses show location of health facilities. Dots show number of ART patients by home village, color-coded by health facility patient attended. Larger dots indicate a larger number of patients.

Active patient enrollment increased from 535 patients at the end of phase 1 to 3,949 patients at the end phase 5 (Table 1, Fig 2). During phases 3 and 4, as additional ART facilities opened, most patients new to these facilities were new ART enrollees. However, 11% of patients transferred during phase 3 (n/N = 257/2,289) and 27% (n/N = 978/3,595) in phase 4. During phase 5 with decentralization complete, the percentage of transfers decreased to 2% (n/N = 91/3,949).

**Table 1:** Demographic, clinical, and distance-to-care for patient cohorts during each phase of full decentralization.

		Phase 1	Phase 2	Phase 3	Phase 4	Phase 5
	Start of phase	1-Aug-06	1-Jun-08	1-Feb-09	1-Aug-10	1-May-12
	End of phase	31-May-08	31-Jan-09	31-Jul-10	30-Apr-12	31-Oct-12
**Demographic information**						
	Health facilities providing ART	1	2	6	12	12
	Number of patients ever enrolled during phase	574	1103	2575	4072	4200
	Number of patients at end of phase^1^ (%)	535 (93)	996 (90)	2289 (89)	3595 (88)	3949 (94)
	Women (%)	359 (67)	651 (65)	1487 (65)	2383 (66)	2623 (67)
	Mean age at ART initiation (sd)	32.5 (13.9)	32.5 (14)	33.2 (14.3)	33 (13.8)	33.2 (13.8)
	Number of transfers during phase (%)^2^	0 (0)	3 (<1)	257 (11)***	978 (27)***	91 (2)***
**Clinical information**						
	Patients who started ART at WHO stage 3 or 4 (%)^3^	389 (73)	630 (63)***	1225 (54)***	1630 (45)***	1748 (44)***
	Median clinic visits per patient per year (IQR)	6.2 (5 to 7)	6.1 (5 to 7)	6.5 (6 to 8)***	7.3 (6 to 9)***	7.6 (6 to 9)***
	Patients with < 4 clinic visits per year (%)	27 (5)	59 (6)	85 (4)	19 (1)***	15 (<1)***
	1-year retention of new patients enrolled during phase (%)^4^	491 (86)	453 (81)	1234 (82)*	1442 (84)	404 (82)
**Distance**						
	Mean travel distance to facility in km (sd)^5^	7.3 (5.4)	7.5 (5.3)	6.3 (4.1)***	4.7 (4.1)***	4.7 (4.1)***
	Patients greater than 8 km (Euclidian) (%)	260 (49)	502 (50)	876 (38)***	717 (20)***	774 (20)***
	Patients attending closest health facility (%)^6^	535 (100)	969 (97)***	1922 (84)***	2117 (59)***	3058(77)***
	Mean cost travel distance in km (sd)^7^	18.8(15.7)	16.6(15.8)*	10.9 (11.9)***	7.8 (9.5)***	7.9(9.6)***

* p < .05,

** p < .01,

*** p < .001;

all comparisons made to Phase 1

^1^ Patients from Neno District enrolled in care at end of phase. All estimates exclude patients receiving care at Neno facilities, but residing outside of Neno District.

^2^ Patients who transferred from one Neno District clinic health facility to another Neno District clinic health facility during phase. The denominator was number of patients ever enrolled during phase.

^3^ 19% of observations were missing WHO stage at ART initiation.

^4^ 1-year retention of all patients who first enrolled at any Neno health facility during the phase.

^5^ Mean Euclidean distance from patient’s home village to health facility where ART care was received. (km)

^6^ A patient attended the nearest health facility if she was enrolled at the facility that was the minimum Euclidean distance from the center of her home village.

^7^ Mean cost distance from patient’s home village to health facility where ART care was received (km)

Mean patient age at ART initiation was 33 years, and approximately two-thirds (64%) of patients were female. There was no evidence that age at initiation or patient gender varied by phase (p > 0.1 for all comparisons). Over time, greater proportions of patients initiated ART for less clinically advanced HIV disease; the percentage of patients who had initiated ART at WHO stage 3 or 4 decreased from 73% in phase 1 to 44% in phase 5 (p < .001).

At the end of phase 1, patients lived an average of 7.3 km from their ART facility, and 49% of patients lived beyond 8 km of their ART facility, not meeting the Malawi MOH’s definition of access to a health facility [26] (Table 1). This percentage decreased significantly over time to 38% at the end of phase 3 (p < 0.001) and 20% at the end of phase 5 (p < 0.001), as average distance to the ART clinic decreased to 4.7 km (p < 0.001). Mean cost distance to ART clinic, which considered topography in the distance calculation, decreased from 18.8 km at the end of phase 1 to 7.9 km at the end of phase 5 (p < 0.001). The proportion of patients with fewer than 4 visits per year ranged from 6% in phase 2 to 1% in phases 4 and 5 (p < .001). Overall, 83% of patients were retained in care for at least one year with minimal variability across phases.

### Transfer analysis

One thousand seventy-four patients (22%) transferred ART facilities at least once (Table 2). Sixty-eight percent (n/N = 731/1,074) of transfers were women. Mean travel distance decreased from 9.5 kilometers to the patient’s pre-transfer facility to 4.7 kilometers for her final facility (p < 0.001), and the percentage of individuals living within 8 km of their health facility increased from 30% to 81% (p < 0.001). Moreover, the proportion of individuals with <4 visits per year decreased from 11% for the initial facility to 1% for the post-transfer facility (p < .001).

**Table 2:** Comparison of demographic, clinical and distance data for transfers and non-transfers.

		Transfers		Non-Transfers
		First Facility	Final Facility^1^	Facility^2^
**Demographic information**				
	Number of patients	1074	1074	3791
	Women (%)	731 (68)	731 (68)	2359 (62)***
	Mean age at ART initiation (sd)	31.8 (14.5)	31.8 (14.5)	33.6 (13.9)***
**Clinical information**				
	Patients starting ART at WHO stage 3 or 4 (%)^3^	557 (52)	557 (52)	1235 (33)***
	Median clinic visits per year (IQR)	6.7 (5.1–9.2)	6.6 (5.8–8.3)	8.3 (6.7–10.5)***
	Patients with <4 visits per year (%)	123 (11)	11 (1)***	45 (1)
	LTFU at 1 year (%)^4^	15 (1)	15 (1)	652 (17)***
**Distance**				
	Mean travel distance to facility, km (sd)^5^	9.5 (4.6)	4.7 (4.4)***	5.1 (4.5)**
	Patients within 8 km (Euclidean) (%)	322 (30)	874 (81)***	2868 (76)***
	Mean cost travel distance in km (sd)^6^	19.7 (16)	9.6 (11.4)***	8.4 (10.8)**

* p < .05,

** p < .01,

*** p < .001

^1^ Asterisks indicate comparisons between transfers at their first facility and the same patients receiving ART at their final facility (repeated-measures ANOVA/Wilcox test for medians)

^2^ Asterisks indicate comparisons between patients who never transferred (non-transfers) and transfers at final facility (ANOVA/Wilcox test for medians)

^3^ Data were missing for 292 transfers at first facility, 229 transfers at final facility, and 1234 non-transfers.

^4^ Patients alive and in care at *any* Neno health facility one year after enrollment

^5^ Mean Euclidean distance from patient’s home village to health facility where ART care was received (km)

^6^ Mean cost distance from patient’s home village to health facility where ART care was received (km)

We also compared patients who experienced at least one transfer (i.e. “transfers”) to those who were only enrolled at one facility during their time in care (i.e. “non-transfers”) (Table 2). Patients who transferred tended to have more severe disease (52% with WHO stage 3 or 4 versus 33% in non-transfers). Only 45 (1%) of non-transfer patients had <4 visits per year, similar to transfer patients at their final facility, also 1.0% per year (p = 0.6). Non-transfers traveled a mean of 5.1 km, similar to the average of 4.7 km traveled by transfers to their final facility (p = .004). However, non-transfer patients were more likely to be LTFU at 1 year (17%) than transfer patients (1%; p < 0.001).

### Distance analysis

In our univariate analysis (n = 6014 enrollment periods of 4865 patients), the hazard ratio associated with living >8 km from a health facility compared to ≤8 km from a health facility was 1.70 (95% CI: 1.5–1.9) (Table 3). In multivariate analysis (Table 3), we controlled for possible confounders, including gender, age, and transfer status. We obtained a nearly identical hazard ratio associated with living >8 km from a health facility (aHR = 1.68, 95% CI: 1.5–1.9). Including WHO stage at ART initiation (n = 3,445) did not substantially impact the estimate of the effect of travel distance on LTFU (aHR = 2.27, 95% CI: 1.8–2.8) (S3 Table).

**Table 3:** Association between travel distance to care and hazard of loss to follow up (LTFU), and exploratory analysis of association between patient covariates and LTFU.

		Unadjusted HR (95% CI)	Adjusted HR (95% CI)^1^
**Travel distance**^2^			
	< 8km	1.00 (Ref)	1.00 (Ref)
	≥ 8km	1.70 (1.51–1.91)***	1.68 (1.49–1.89)***
**Age (years)**^3^		1.00 (1.00–1.01)	1.00 (0.995–1.00)
**Gender**			
	Female	1.00 (Ref)	1.00 (Ref)
	Male	1.64 (1.46–1.84)***	1.62 (1.44–1.82)***
**Transfer**^4^			
	No	1.00 (Ref)	1.00 (Ref)
	Yes	1.05 (0.87–1.28)	1.11 (0.91–1.34)

Cox proportional hazards regression (n = 6014 enrollment periods of 4865 patients): primary outcome variable was loss to follow up. Travel distance and transfer were treated as time-varying covariates; other covariates remained constant over time.

* p < .05,

** p < .01,

*** p < .001

^1^ Adjusted for all other variables in table

^2^ Euclidean distance from patient’s home village to health facility where ART care was received; measured as a time-varying covariate based on a patient’s location during a particular time interval

^3^ Age at ART initiation, centered at mean

^4^ Compares patients who had transferred from one Neno health facility to another to those at their first facility, also time-varying.

## Discussion

Employing the first application of geospatial data, to our knowledge, to quantify the relationship between patient-level travel distance to ART services and patient outcomes, we examined the impact of ART decentralization on travel distance, appointment adherence, and LTFU in a rural setting in SSA. We observed that ART decentralization was associated with increased patient enrollment, reduced patient travel distance, and decreased LTFU.

Though HIV testing and counselling (HTC) was available at all Neno health facilities beginning in mid-2007, many patients meeting ART eligibility criteria did not begin ART until their local facility offered it. In 2008, the patient home village distribution was highly concentrated around the only facility offering ART. By 2012, when ART was available at all 12 facilities in the district, the home village distribution of ART patients corresponded roughly to the general population distribution. Furthermore, 80% of patients in Neno traveled less than 8 km to reach their chosen facility. Among patients who transferred to a closer facility after having previously received ART at another facility located further away, the percentage with poor appointment adherence decreased from 11% to 1% after transfer. Proximity to ART care appeared to be a key driver for treatment initiation and adherence in this rural African population with little access to motorized transportation.

Previous studies from Malawi have demonstrated conflicting results about the effects of ART decentralization on patient outcomes. In rural Thyolo District, although the incidence of patient LTFU was significantly lower for patients started on ART at primary health facilities compared to those initiating ART at the district hospital, ART decentralization was associated with higher patient mortality [11]. In Zomba District, patients receiving decentralized care were 40% less likely to experience LTFU and death during the 10 month study period but the study included a relatively healthy population [29]. Our results suggest that full ART decentralization for all patients can achieve favorable results in Malawi and similar impoverished rural SSA regions, and may help achieve the ART provision and viral suppression targets of the 90-90-90 UNAIDS Declaration.

We detected differences in LTFU between ART enrollees who transferred facilities, often enrolling in earlier phases, and later enrollees who initiated ART once a nearby facility began offering treatment. Eighty-three percent of patients who did not transfer were retained in care for at least 1 year, compared to 99% of transfers, despite similar average travel distances. We hypothesize that patients who enrolled in ART earlier, when ART facilities were more limited, were generally a more adherent population. Nevertheless, we found strong evidence that shorter travel distance was associated with lower LTFU for both transfers and non-transfers.

The major strength of this study resides in our ability to examine the longitudinal relationship between individual patients’ distance to care and ART treatment outcomes. Previous research has typically divided patients into those receiving care at centralized facilities and those receiving care at decentralized facilities, without the ability to detect patient-level variations in travel time [6,29,30]. While some studies have investigated travel time for HIV patients [31,32], these either did not examine patient outcomes, or found little relationship between travel time and appointment adherence. By contrast, we observed a strong association between distance to care and LTFU.

Our study had several limitations. First, guideline changes and other secular trends during the study period may have introduced bias. For example, in 2011, the Option B+ strategy was introduced nationally resulting in increased ART enrolment of relatively healthy individuals. In addition, anecdotal evidence suggests that HTC uptake increased in Neno as ART services became more widely available in newly ART-accredited facilities. As a result, the number of patients eligible to start ART significantly increased over time, and we were unable to disentangle how decentralized care affected ART enrollment after accounting for these factors. Second, changes in the way PIH/APZU provided HIV care during the study period may have influenced enrolment, adherence, and retention in care. For example, at ART initiation patients received a supplemental food package and the duration of this food support changed during the study period. We cannot predict the magnitude or direction of the effects of these changes in HIV care on our study outcomes; however, the relatively stable one-year retention proportion of 81–86% over the 5 study phases suggests that these effects may have been minimal. Third, missing clinical information, such as baseline WHO stage, limited our ability to track changes in our cohort over time, particularly with regard to HIV disease severity and immunosuppression at ART initiation. Fourth, because Malawi has limited data on subnational HIV trends [33], we were unable to consider ecological trends in HIV prevalence in Neno.

Our geospatial analysis also had some limitations. As each village had only one GPS point, we could only approximate each patient’s home location, and because the electronic medical record did not always denote patient address changes, we were unable to detect all patient moves. Village data were also missing for approximately 5% of patients. Euclidean distances do not represent the true distance patients travel to receive care given that footpaths and roads adapt to terrain. The use of cost distance corrects for elevation changes, but likely still underestimates the true distance traveled by patients to reach their health facility. Finally, we were unable to identify patients who initiated and maintained ART at mobile clinics prior to the opening of the nearest decentralized ART clinic, which may have overestimated travel distance.

Nevertheless, we believe that the results of our study are generalizable to other rural settings in Malawi and elsewhere in SSA. We observed a strong association between ART decentralization, decreased patient travel distance, and increased ART enrollment and retention in care. Our data, derived from a public sector ART program, reflect that full ART decentralization can improve outcomes even under “real-world” clinical conditions in an extremely impoverished, rural district. The use of GIS technology and mathematical modeling can further support the rational and equitable introduction of additional ART sites in Malawi [34]. As care for other chronic illnesses is scaled up and decentralized in SSA, it will be useful to assess geographic access to care and its relationship to treatment adherence and other clinical and service-related outcomes.

In conclusion, our findings highlight the benefits of decentralized ART care and the potential of increasingly robust geographic information systems to improve our understanding of how distance to health facilities affects LTFU and other outcomes for HIV-infected patients.

## Supporting information

S1 Table Comparison between patients with and without village information. (DOCX)

S2 Table Association between cost distance to care and hazard of loss to follow up (LTFU), and exploratory analysis of association between patient covariates and LTFU. (DOCX)

S3 Table Sensitivity analysis of relationship between travel distance to care and hazard of loss to follow up (LTFU). (DOCX)
