# Supplementary material for: Distance to care, enrollment and loss to follow-up of HIV patients during decentralization of antiretroviral therapy in Neno District, Malawi: A retrospective cohort study
Source: PLoS One. 2017 Oct 3;12(10):e0185699. doi: 10.1371/journal.pone.0185699 (PMC5626468; doi:10.1371/journal.pone.0185699)
Supplement: S1 Table — (DOCX) [file pone.0185699.s001.docx]

**S1 Table. Comparison between patients with and without village information.**

|  | **Had village data (n=5668)** | **Missing village data (n = 301)** | **P-value** |
| --- | --- | --- | --- |
| **Women (%)** | 3606 (64) | 166 (55) | ** |
| **Mean age at ART initiation (sd)** | 33.4 (14) | 34.8 (13.4) |  |
| **WHO stage of 3 or 4 at initiation (%)** | 2116 (37) | 97 (32) |  |
| **Median clinic visits per year (IQR)** | 8 (6 to 10) | 9.3 (7 to 13) | *** |
| **Patients with <4 clinic visits per year (%)** | 66 (1) | 5 (2) |  |
| **Patients in care ≥1 year (%)** | 4673 (82) | 194 (64) | *** |

Patients were considered as having village information if at least one record contained a valid village, either inside or outside of Neno District.

* p < .05, ** p<.01, *** p < .001
