# Supplementary material for: Distance to care, enrollment and loss to follow-up of HIV patients during decentralization of antiretroviral therapy in Neno District, Malawi: A retrospective cohort study
Source: PLoS One. 2017 Oct 3;12(10):e0185699. doi: 10.1371/journal.pone.0185699 (PMC5626468; doi:10.1371/journal.pone.0185699)
Supplement: S2 Table — (DOCX) [file pone.0185699.s002.docx]

**S2 Table. Association between cost distance to care and hazard of loss to follow up (LTFU), and exploratory analysis of association between patient covariates and LTFU.**

|  |  | **Unadjusted OR**  **(95% CI)** | **Adjusted OR**  **(95% CI)^1^** |
| --- | --- | --- | --- |
| **Cost distance^2^** |  |  |  |
|  | < 8km | 1.00 (Ref) | 1.00 (Ref) |
|  | ≥ 8km | 1.26 (1.13-1.42)*** | 1.26 (1.12 -1.41)*** |
| **Age (years)^3^** |  | 1.00 (1.00-1.01) | 1.00 (0.99-1.00) |
| **Gender** |  |  |  |
|  | Female | 1.00 (Ref) | 1.00 (Ref) |
|  | Male | 1.64 (1.46-1.84)*** | 1.64 (1.46-1.84)*** |
| **Transfer^4^** |  |  |  |
|  | No | 1.00 (Ref) | 1.00 (Ref) |
|  | Yes | 1.05 (0.87-1.28) | 1.06 (0.87-1.28) |

Cox proportional hazards regression (n = 6014 enrollment periods of 4865 patients): primary outcome variable was loss to follow up. Cost distance and transfer status were treated as time-varying covariates; other covariates remained constant over time.

** p < .05, ** p<.01, *** p < .001*

^1^ Adjusted for all other variables in table

^2^ Cost distance based on topological slope from patient’s home village to health facility where ART care was received; measured as a time-varying covariate based on a patient’s location during a particular time interval

^3^ Age at ART initiation, centered at mean

^4^ Compares patients who had transferred from one Neno health facility to another to those at their first facility, also time-varying.
