# Supplementary material for: Distance to care, enrollment and loss to follow-up of HIV patients during decentralization of antiretroviral therapy in Neno District, Malawi: A retrospective cohort study
Source: PLoS One. 2017 Oct 3;12(10):e0185699. doi: 10.1371/journal.pone.0185699 (PMC5626468; doi:10.1371/journal.pone.0185699)
Supplement: S3 Table — (DOCX) [file pone.0185699.s003.docx]

**S3 Table. Sensitivity analysis of relationship between travel distance to care and hazard of loss to follow up (LTFU).**

|  |  | **Adjusted OR**  **(95% CI)**  **LTFU: 60 days^1^** | **Adjusted OR**  **(95% CI)**  **LTFU: 90 days^2^** | **Adjusted OR**  **(95% CI)**  **LTFU: 180 days + transfer interaction^3^** | **Adjusted OR**  **(95% CI)**  **LTFU: 180 days +**  **WHO stage** |
| --- | --- | --- | --- | --- | --- |
| **Travel distance^5^** |  |  |  |  |  |
|  | < 8km | 1.00 (Ref) | 1.00 (Ref) | 1.00 (Ref) | 1.00 (Ref) |
|  | ≥ 8km | 1.43 (1.29-1.59)*** | 1.64 (1.47-1.84)*** | 1.66 (1.46 -1.87)*** | 2.27 (1.82-2.84)*** |
| **Age (years)^5^** |  | 1.00 (0.996-1.003) | 1.00 (0.99-1.00) | 1.00 (0.995-1.003) | 1.00 (0.99-1.00) |
| **Gender** |  |  |  |  |  |
|  | Female | 1.00 (Ref) | 1.00 (Ref) | 1.00 (Ref) | 1.00 (Ref) |
|  | Male | 1.48 (1.35-1.64)*** | 1.59 (1.42-1.77)*** | 1.62 (1.44-1.82)*** | 1.33 (1.06-1.67)* |
| **Transfer^6^** |  |  |  |  |  |
|  | No | 1.00 (Ref) | 1.00 (Ref) | 1.00 (Ref) | 1.00 (Ref) |
|  | Yes | 0.84 (0.72-0.97)* | 1.02 (0.86-1.22) | 1.06 (0.85-1.34) | 0.91 (0.62-1.32) |
|  |  |  |  |  |  |
| **Transfer***  **travel distance** |  |  |  |  |  |
|  | Non-transfer OR < 8km | - | - | 1.00 (Ref) | - |
|  | Transfer AND ≥ 8km | - | - | 1.13 (0.78-1.65) | - |
|  |  |  |  |  |  |
| **WHO stage at ART initiation** |  |  |  |  |  |
|  | Stage I | - | - | - | 1.00 (Ref) |
|  | Stage II | - | - | - | 0.94 (0.66-1.34) |
|  | Stage III | - | - | - | 0.62 (0.48-0.80)*** |
|  | Stage IV | - | - | - | 0.80 (0.50-1.28) |

Cox proportional hazards regression: primary outcome variable was loss to follow up. Cost distance and transfer status were treated as time-varying covariates; other covariates remained constant over time.

* p < .05, ** p<.01, *** p < .001

^1^ Adjusted for all other variables in table; outcome variable (LTFU) defined as having vital status was recorded as dead, defaulted, stopped or transferred out (without a corresponding “transfer in” to a different facility) or if the patient did not have ≥1 recorded ART visit with 60 days of the December 30, 2013 (n = 6014 enrollment periods of 4865 patients)

^2^ Adjusted for all other variables in table; outcome variable (LTFU) defined as having vital status was recorded as dead, defaulted, stopped or transferred out (without a corresponding “transfer in” to a different facility) or if the patient did not have ≥1 recorded ART visit with 90 days of the December 30, 2013 (n = 6014 enrollment periods of 4865 patients)

^3^ Adjusted for all other variables in table; outcome variable (LTFU) defined as having vital status was recorded as dead, defaulted, stopped or transferred out (without a corresponding “transfer in” to a different facility) or if the patient did not have ≥1 recorded ART visit with 180 days of the December 30, 2013 (n = 6014 enrollment periods of 4865 patients)

^3^ Adjusted for all other variables in table; outcome variable (LTFU) defined as having vital status was recorded as dead, defaulted, stopped or transferred out (without a corresponding “transfer in” to a different facility) or if the patient did not have ≥1 recorded ART visit with 180 days of the December 30, 2013 (n = 4251 enrollment periods of 3445 patients)

^5^ Euclidean distance from patient’s home village to health facility where ART care was received; measured as a time-varying covariate based on a patient’s location during a particular time interval

^6^ Age at ART initiation, centered at mean

^7^ Compares patients who had transferred from one Neno health facility to another to those at their first facility, also time-varying.
